# Plant viruses traveling without passport

**DOI:** 10.1371/journal.pbio.3002626

**Published:** 2024-05-10

**Authors:** Cristina Sáez, Israel Pagán

**Affiliations:** Centro de Biotecnología y Genómica de Plantas UPM-INIA and E.T.S. Ingeniería Agronómica, Alimentaria y de Biosistemas, Universidad Politécnica de Madrid, Madrid, Spain

## Abstract

All plant viruses were thought to encode in its genome a movement protein that acts as a “passport”, allowing active movement within the host. This primer highlights a study that characterizes the first plant virus that can colonize its host without encoding this protein.

## Teaser

All plant viruses were thought to encode in its genome a movement protein that acts as a “passport,” allowing active movement within the host. A new study in *PLOS Biology* characterizes the first plant virus that can colonize its host without encoding this protein.

Viruses are the main causal agents of emerging diseases in plants [1]. Recent examples are the rapid spread of cucumber green mottle mosaic virus in cucurbit crops worldwide [2], or tomato yellow leaf curl disease, caused by a complex of plant viruses, which affects tomato crops globally [3]. These epidemics are still to be efficiently controlled, causing significant yield losses, and representing a continuous threat for food safety [2,3]. To reduce such negative impacts, it is essential to gain deep knowledge on how plant viruses achieve successful infections, for which they need to colonize the plant from entry points.

**Fig 1:**
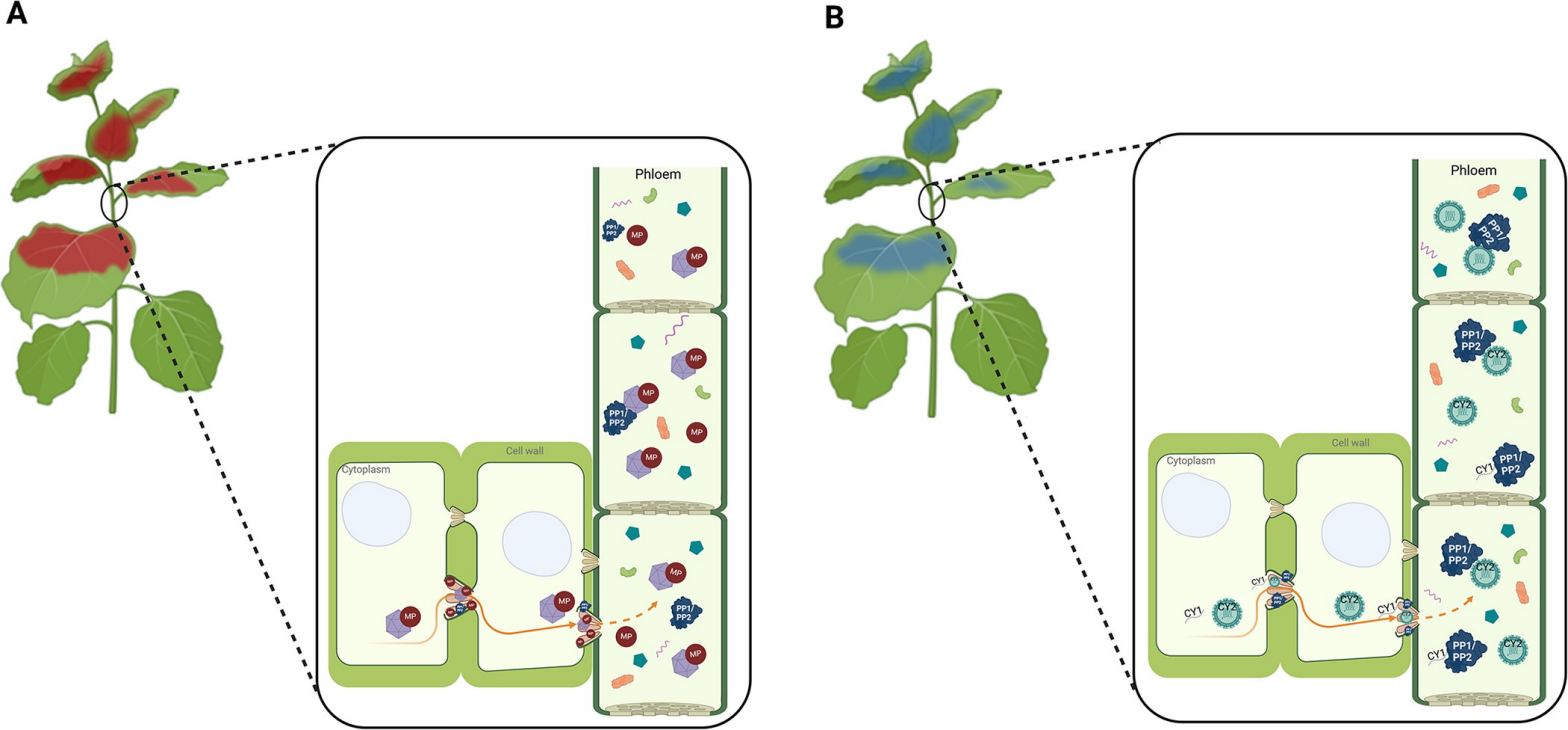
Schematic representation of plant virus within-host movement. (A) Viral MP-dependent movement, as required for most plant viruses, in which host proteins (phloem proteins 1 and 2, and/or others) may also be involved. Movement may involve virions (purple hexagons), but for most RNA viruses it involves naked RNA protected from RNases by viral and/or host proteins (ribonucleoprotein complexes) like citrus yellow vein associated virus 1 (CY1) in the adjacent panel. (B) Host MP-dependent movement as shown by Ying and colleagues [5] for CY1 and CY2, which depends on the host protein PP2. Viral (red dots) and host (dark blue shapes) MPs act as “passports” for viral cell-to-cell and long-distance movement through the phloem. Viruses can move as virions (light blue spheres) or as naked genomes (wavy purple lines) that are protected by host proteins like PP2. For clarity, naked CY1 viral RNA is shown as attached to a single PP1/PP2 protein, but these proteins cover the entire molecule to exert their protective role. Phloem also transports other molecules: small RNAs (wavy pink lines), small proteins (green shapes), sugars (blue pentagons), etc., which move freely, and large molecules (orange ovals) that require active transport like viruses. Scheme was constructed using BioRender.

Unlike animal viruses, which evolved receptor-mediated mechanisms to facilitate exploiting the extracellular environment for within-host dissemination, plant viruses are restricted to the intracellular compartment (symplast). Thus, cell-to-cell movement occurs through symplastic channels (plasmodesmata), which transverse the plant cell walls and connect adjacent cells. The plasmodesmata are not cell-to-cell highways: Although they are permeable for small molecules, there is a size exclusion limit that precludes free passage of larger molecules such as virus particles [4]. In these cases, active transport mechanisms are required, and plant virus genomes encode movement proteins (MPs) that act as “passports” by increasing the size exclusion limit and mediating virus passage across plasmodesmata. This way, viruses reach the plant vasculature, but they still need to transit through it to colonize other parts of the plant. For that, plant viruses are mainly transported via phloem—the living tissue in vascular plants that transports the soluble organic compounds made during photosynthesis—, a process in which MPs are also very important [4]. Thus, MPs play crucial roles in short- and long-distance dissemination of plant viruses within the host (**Fig 1**), and it has been long accepted that MPs are their signature proteins. The work by Ying and colleagues [5] in this issue identifies 2 plant viruses (citrus yellow vein associated virus 1 and 2: CY1 and CY2) that systemically colonize their host without needing an MP, which may represent a game-changer in the current view of how these pathogens move within their hosts and achieve successful infections.

CY1 and CY2 are not the only plant viruses that lack MPs. Then, what makes them so unique? Currently, there are 2 known types of plant viruses lacking MPs: (i) viruses that are strictly transmitted from parent-to-offspring via seeds [6], such as species in the families *Endornaviridae*, *Narnaviridae*, and *Mitoviridae*. Because these viruses only need to invade the plant germline, which they do via cell division, cell-to-cell movement is not needed making MPs unnecessary [7]. (ii) Plant viruses that are not self-sufficient and, because they lack MP, need to partner with another virus that provides this function. For instance, members of the genus *Umbravirus* associate with species of the genus *Luteovirus* as helper viruses using their MPs for within-host colonization. In contrast, no helper viruses have been identified for CY1 and CY2. Moreover, although it is not clear how these viruses are dispersed in nature, it has been proposed that they could be transmitted by insect vectors, and they are experimentally transmitted via grafting of infected plant material into healthy one [8]. This striking discovery therefore shows that, first, harboring an MP is not mandatory for plant viruses, which changes the current view of the features that constitute the identity of these pathogens, and second, that the minimum protein toolkit that plant viruses need to complete their life cycle (which is typically considered to be restricted to a replication protein, an MP, an RNA silencing suppressor, and a capsid protein [9]) can be even smaller. This highlights the extraordinary capacity of plant viruses to subvert the host functioning with even less proteins that was thought to date.

If CY1 and CY2 do not require an MP for host colonization, how do they do it? During infection, plant viruses significantly alter protein composition within cells and in the phloem, both because of the plant defence response and of virus manipulation of the host machinery to its benefit. Then, it is logical to think that at least part of these changes would help the pathogen to achieve a successful infection, for instance, contributing to plant colonization. Indeed, several plant proteins have been described to facilitate virus short- and long-distance within-host dissemination [4]. The role of these host proteins is not mutually exclusive with that of MPs and has been traditionally considered to be necessary but not sufficient for virus movement. Starting from the hypothesis that, in the case of CY1 and CY2, host proteins may complement the lack of MPs, Ying and colleagues [5] identify phloem protein 2 (PP2) as necessary for plant colonization (**Fig 1**). The PP2, together with PP1, is the most abundant phloem protein in cucurbits [10]. Both have structural characteristics featured by MPs, modify the exclusion size of plasmodesmata and appear to protect the virus during phloem trafficking, being considered as plant MPs. Although PP1 and PP2 has been reported to interact with various plant viruses even if these encode their own MPs [4], this is the first time that it is shown that they can replace the function of viral MPs. This suggests that PP1 and PP2 play a main role in the life cycle of a wide range of plant viruses. Future studies should confirm this idea and, if so, these phloem proteins will be ideal candidate targets to develop control strategies based on interfere with virus within-host colonization.

Relying on a host protein for a crucial step in the virus life cycle such as within-host colonization, rather than encoding the required function in its own genome, seems to be a risky strategy for CY1 and CY2. Therefore, how can we explain the evolution of these viruses? A plausible explanation is that they do not represent an evolutionary end point but an intermediate. It has been proposed that plant viruses evolved from insect viruses by acquiring MPs in 2 different ways: by incorporating in their genomes the MPs from the host plant or by duplicating the coat protein (CP) and repurposing one copy to act as MP. This second hypothesis is based on the structural similarities of CPs and many MPs, and recent work based on computer protein folding supports it [11]. Interestingly, CY2 encodes a protein with structural characteristics common to MPs and CPs. Ying and colleagues [5] show that this protein is not an MP, but rather it would act as a CP. This would be compatible with the idea that CY2 would be an intermediate of evolution from which an MP may evolve by duplication and repurposing of the CP. Further analyses are needed to address this question, but certainly the characterization of CY1 and CY2 provide new tools to understand the origins of plant viruses either through phylogenetic studies or experimental evolution.
